# Changes in Motor Performance and BMI of Primary School Children over Time–Influence of the COVID-19 Confinement and Social Burden

**DOI:** 10.3390/ijerph19084565

**Published:** 2022-04-10

**Authors:** Stefanie Wessely, Nina Ferrari, David Friesen, Martin Grauduszus, Marlen Klaudius, Christine Joisten

**Affiliations:** 1 Department for Physical Activity in Public Health, Institute of Movement and Neurosciences, German Sport University Cologne, Am Sportpark Müngersdorf 6, 50933 Cologne, Germany; nina.ferrari@uk-koeln.de (N.F.); d.friesen@dshs-koeln.de (D.F.); m.grauduszus@dshs-koeln.de (M.G.); m.klaudius@dshs-koeln.de (M.K.); c.joisten@dshs-koeln.de (C.J.); 2 Cologne Center for Prevention in Childhood and Youth, Heart Center Cologne, University Hospital of Cologne, Kerpener Str. 62, 50937 Cologne, Germany

**Keywords:** COVID-19-confinement, BMI, motor performance, social burden, children

## Abstract

During the COVID-19 pandemic, physical inactivity and being overweight increased in children, especially those from lower socioeconomic backgrounds. Additionally, a decrease in motor performance has been increasingly reported; however, there is a lack of meaningful data on the relationship between these findings and socioeconomic status. Therefore, we examined the BMI, BMI *z* score, the prevalence of overweight/obesity, and motor performance (based on lateral jumping, the standing long jump, and the 6-min run) among three cohorts from timelines before (2016) and during the pandemic (2020, 2021), using a cohort sequential design. Our analysis revealed that all parameters examined showed a significant negative trend over time. For children with a low social burden, the trend was statistically detectable only for lateral jumping and the 6-min run. For children with a high social burden, the prevalence of overweight/obesity increased from 4.2% to 5.4% between 2016 and 2021. The prevalence of being underweight also increased from 3.4% to 4.3% during this time. Motor performance decreased from 11.3% to 12.3%, except in the standing long jump. Children with a high social burden showed significantly lower results (7.4% to 9.6%). Thus, our data confirm an increase in both measurements of overweight/obesity and underweight, as well as a decrease in motor performance under the pandemic conditions, especially in children from socioeconomically deprived backgrounds. These findings affirm the urgent need for action regarding health promotion measures, especially for children with high social burdens.

## 1. Introduction

Physical activity and fitness have indisputable health benefits for children and adolescents [1,2]. However, a significant decline in children’s physical activity and cardiovascular and motor fitness has been observed in recent decades, especially among primary school children [3,4,5]. Accordingly, the prevalence of being overweight and obese has increased or remained high in Germany [6]. Since the onset of the COVID-19 pandemic, this negative trend has been exacerbated by school closures, the canceling of physical education classes and distance learning, closures of public sports facilities, and the lack of organized sports opportunities [7]. In the meantime, numerous studies have shown a predominant reduction in physical activity in childhood and adolescence [8]. A review by Rossi et al. [9] described a decline of 45–91 min per day compared to prior to the pandemic. However, the data are inconsistent and depend, among other things, on the design of the studies, which were primarily cross-sectional analyses and surveys. In Germany, based on data from the Motorik-Modul (MoMo) study, daily physical activity first increased from 108.0 to 146.8 min per day during the first lockdown (March–June 2020), then decreased to 62.2 min during the second lockdown (November 2020–January 2021) [10,11,12]. Other studies, noting similar results, concluded that these trends were largely influenced by environmental and/or socioeconomic conditions [9,11,13,14]. For example, Rundle et al. [15] found that the impact of COVID-19 restrictions on physical activity was more severe in children living in urban areas without access to safe, accessible outdoor spaces than it was for those living in areas with these amenities. Families with a lower socioeconomic status (SES) had less access to outdoor space and consequently, their children had fewer opportunities for physical activity [16]. Similarly, children with access to green spaces showed the greatest increase in physical activity during the first lockdown, according to the MoMo study [11].

Unsurprisingly, during the lockdown, children spent extended periods engaged with digital media, regardless of their socioeconomic status—although children with lower SES consumed more compared to peers with higher SES [11]. Various studies have shown an increase in media use of 1 to 4 h a day [10,17,18], and in obese children, this number rose to up to 5 h per day [19]. In general, less physical activity and more screen time are associated with obesity, cardiometabolic risk factors, poor physical performance and worse mental health, and this association was even stronger during the COVID-19 pandemic [20,21,22].

To date, however, there are few objectively measured data assessing the effects of motor and cardiovascular fitness on primary school-aged children, which has also provided conflicting results. Using the 20-m-shuttle-run test (among other tests), Chambonniere et al. [23] noted a reduction in maximal oxygen uptake in 206 children aged 9.9 (1.0) years—from 45.7 (4.3) pre-pandemic to 43.1 (3.7) mL/min/kg peri-pandemic, as well as a 34.8 cm reduction in standing long jump performance. Jarning et al. [24] described a decline in the 6-min run by 764 children aged 7–10 years from 917.0 (141.1) pre-pandemic to 815.0 (134.3) m peri-pandemic. Basterfield et al. [25] found a reduction from 23 (13.6) to 21 (12.6) total shuttle numbers within the 20-m-shuttle-run test and an increase of 122.0 (23.0) to 129.0 (24.0) cm in the standing long jump in English children aged 9.1 (0.6) years from pre- to peri-pandemic.

Tsoukos et al. [26] described the reduced physical performance in several fitness tests and a higher body mass index (BMI) in youth experiencing COVID-19 lockdown (2020–2021) compared with pre-pandemic cohorts (2016–2017 and 2018–2019). Basterfield et al. [25] also found a 1.5 kg/m^2^ increase in BMI. Chang et al. [27] conducted a meta-analysis and reported a mean increase in body weight of 2.7 kg and an increase in BMI of 0.8 kg/m^2^ over a period of 1–4 months after the onset of lockdowns. Woolford et al. [28] compared 191,509 children from two cohorts (March 2019–January 2020 vs. March 2020–January 2021) in the Kaiser Permanente Southern California electronic health record database; they described a 1.6 kg/m^2^ increase in BMI among children 5–11 years old.

Despite this body of research, the extent to which the socioeconomic disparities mentioned earlier are reflected in the motor performance of primary school children during, compared to before the pandemic, is still largely unclear.

Therefore, in the present study, we considered anthropometric data and selected motor performance benchmarks of primary school children in different cohorts, comparing the results before (2016) and during (2020 and 2021) the COVID-19 pandemic and as a function of socioeconomic background.

## 2. Materials and Methods

### 2.1. Study Population

**Figure 1:**
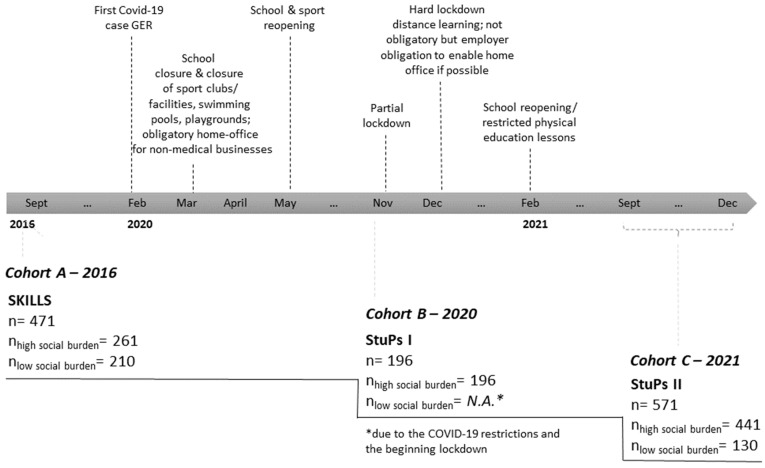
Flow chart of the data sources considering pandemic events.

This study used a cohort sequential design. Three cohorts were considered (Figure 1): Cohort A originated from testing within the SKILLS project [29] conducted in 2016, while Cohorts B and C originated from testing within the StuPs project [30] conducted in 2020 (Cohort B) and 2021 (Cohort C), under pandemic conditions. The different COVID-19 confinements according to the different time points are shown in Figure 1. Data from a total of 1238 children were integrated into this analysis (*n*_Cohort A 2016_ = 471, *n*_Cohort B 2020_ = 196, *n*_Cohort C 2021_ = 571). Both studies obtained ethical approval according to the Declaration of Helsinki. In both studies, consent forms were distributed to all children in the participating schools, and all children who could provide signed consent from their parents were included in the studies.

Cohort A comprised data from 261 children from schools with high social burdens, along with 210 children from schools with low social burdens. Cohort B included 196 datasets from children from schools with high social burdens. Cohort C contained data on 441 children from schools with high social burdens and 130 with low social burdens (Table 1, Figure 1). The research methodology used for Cohort B differed slightly from that of the other time points due to the COVID-19 measures in effect at that time. Immediately as testing in schools with high social burdens commenced, the second lockdown began in Germany, thereby preventing testing in additional schools with low social burdens. Therefore, the analyses partially include only Cohort A and C. An overview of all variables examined can be found in Table A1.

Differences between children with high social burdens in the three Cohorts (A, B and C) were analyzed first. Subsequently, Cohort A and Cohort C were compared to assess differences according to low and high social burdens.

### 2.2. Analysis Parameter

#### 2.2.1. Anthropometric Data

The children’s height (cm) and weight (kg) were measured barefoot using a calibrated stadiometer and scale in a standardized manner, as described elsewhere [31]. Body mass index (BMI kg/m^2^) was calculated. These values were then categorized, with those above the 90th percentile considered overweight and those above the 97th percentile considered obese according to German norm values by Kromeyer-Hauschild [32], and as further described elsewhere [32,33].

In addition, the BMI standard deviation score (BMI *z* score) was calculated using the least mean squares (LMS) method for non-normally distributed characteristics [24,34]:


$$z\;{\mathrm{score}}_{\mathrm{LMS}}=\frac{{\left[\mathrm{BMI}/M[t]\right]}^{L[t]}-1}{L[t]S[t]}$$

M[t], L[t], and S[t] are parameters for the participant’s age [t] and sex.

#### 2.2.2. Motor Test Items: Lateral Jumping, Standing Long Jump, 6-min Run

Motor performance was assessed using selected items from the Dordel–Koch test (DKT) [5]. A detailed description of the test battery as well as background information regarding the definition of motor performance has been described elsewhere [5]. Of the seven test items, we assessed lateral jumping, the standing long jump, and the 6-min run. Agility and coordination were determined based on the lateral jumping item. For this purpose, lateral jumps with both legs crossing a line simultaneously were performed in two rounds of 15 s each, and the number of jumps from both rounds was summed. The standing long jump item was performed to determine lower-extremity power. For this purpose, the best of two two-legged jumps from standing was measured in centimeters. To assess cardiorespiratory and aerobic endurance capacity, the participants completed a 6-min run, trying to reach as many meters as possible within 6 min. This test item was only used in Cohorts A and C; it could not be included in testing Cohort B due to COVID-19 safety precautions. Motor testing was performed in a standardized manner by previously trained test administrators, as described elsewhere in detail [5].

#### 2.2.3. Socio-Cultural Variable: School-Based Social Index

The children’s social burden was determined using a central index for schools: the school-based social index (“Schulscharfer Sozialindex”) from North Rhine-Westphalia, in Germany [35]. This index is based on the following four indicators:

▪ Children and youth poverty-assessed by the proportion of children living in the vicinity of the primary school with parents receiving governmental financial support for jobseekers (SGB II rate);
▪ The proportion of children in families whose primary language is not German;
▪ The proportion of children who immigrated from abroad;
▪ The proportion of children with special needs in learning, emotional and social development, or language.

Using confirmatory factor analyses, the authors merged these four indicators and assigned scores on a social index scale ranging from 1–9, where level 1 corresponds to a very low burden and level 9 indicates a very high burden (ibid.). Our study population spanned social index levels 1–7, with an average of 5.0 (2.1). In the following analysis, the social index was used as a dichotomous variable that categorizes index levels 1–5 as a low social burden and levels 6–9 as a high social burden. This allocation was made in alignment with the statistical data of the City of Cologne, which defines a social index for each city district consisting of information on the following parameters: the district’s degree of economic, politico-cultural, and health disadvantages [36].

### 2.3. Data Analysis

Data analysis was performed using SPSS Statistics 28.0 software (IBM Corp., Armonk, NY, USA). For descriptive statistics, we calculated mean value and standard deviation (SD) for the metric variables and calculated frequency distributions for the categorical variables for all measurement time points. We applied univariate analysis of variance (ANCOVA) to determine group differences among the three Cohorts (A, B, and C) solely for those children from schools with high social burdens for the motor test items and anthropometric data. Analysis of anthropometric data was adjusted for age and sex. Analysis of motor test items was adjusted for age, sex, and BMI. Two-factor analyses of variance with covariates were performed to assess the differences between the variances in Cohorts A and C as well as the social index regarding the motor test items and anthropometric data. Analysis of anthropometric data was adjusted for age and sex. Analysis of motor test items was adjusted for age, sex, and BMI. Post hoc tests with Bonferroni correction were used to calculate the differences between the groups. The significance level for all tests was 5%. The corrected coefficient of determination (R^2^) was used to interpret the results, along with the proportion of the variance of the dependent variable explained by the overall model, while the respective partial eta squared (n_p_^2^) was used for the covariates.

## 3. Results

### 3.1. Anthropometric Data–Descriptive Presentation

The average age of the total population was 8.1 (1.2) years and 51.6% were male. On average, participants were 130.9 (9.1) cm tall, weighed 30.6 (8.3) kg, and had a mean BMI of 17.6 (3.2) kg/m^2^, or a BMI *z* score of 0.5 (1.1). Thus, 71.3% were normal weight, 4.1% were underweight, 13.1% were overweight, and 11.5% were obese (Table 1).

For high social burden, BMI increased from 17.4 kg/m^2^ in Cohort A to 18.3 kg/m^2^ in Cohort B and 18.2 kg/m^2^ in Cohort C, while the BMI *z* score increased from 0.4 (1.0) in Cohort A to 0.6 (1.3) in Cohort B and 0.7 (1.2) in Cohort C. The proportion of normal-weight children decreased from 73.9% in Cohort A to 66.3% in Cohort B and 63.5% in Cohort C, whereas the proportions of overweight and obese children increased from 11.9% and 10.7% in Cohort A to 17.3% and 12.8% in Cohort B and 16.1% for each category in Cohort C. The proportion of underweight children also increased from 3.4% in Cohort A to 3.6% in Cohort B and 4.3% in Cohort C (Table 1).

For low social burden, BMI increased from 16.7 kg/m^2^ in Cohort A to 16.9 kg/m^2^ in Cohort C; the BMI *z* score did not change, remaining at 0.2 (0.9) in both Cohorts A and C; 85.7% of Cohort A were normal weight, compared with 76.9% of children in Cohort C. The proportions of overweight and obese changed from 5.2% and 5.7% in Cohort A to 11.5% and 4.6% in Cohort C. The proportion of underweight children was 3.3% in Cohort A and 6.9% in Cohort C (Table 1).

#### 3.1.1. Cohort Comparisons of BMI and BMI *z* Score (High Social Burden)

**Figure 2:**
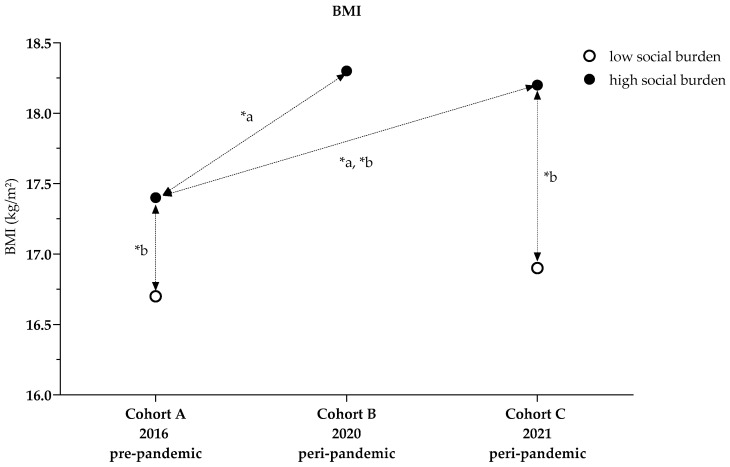
BMI comparisons between cohorts and influenced by social index; * a *p*-values ANCOVA adjusted for age and sex, Table A2; * b *p*-values two-factor analysis adjusted for social index, age, sex, Table A3 and Table A4, Model 1.

The univariate variance model with the dependent variable BMI showed that children in Cohort A had lower BMIs compared with the other cohorts (*p* < 0.001). Children in Cohort B had BMIs that were on average 4.9% higher than those in Cohort A (+0.8 (0.3) kg/m^2^, *p* = 0.020), while children in Cohort C had BMIs 5.4% higher than those in Cohort A (+0.9 (0.3) kg/m^2^, *p* < 0.001). Cohorts B and C did not differ. Age (*p* < 0.001, n_p_^2^ = 0.044), but not sex affected the BMI. The overall model explained 5.8% of the variance (R^2^ = 0.058) (Figure 2, Table A2).

The univariate variance model with the dependent variable BMI *z* score showed that children in Cohort A had lower BMI *z* scores than those in Cohorts B and C (*p* = 0.005). In Cohort C, the average BMI *z* score was higher than in Cohort A (+0.3 (0.1), *p* < 0.001). Cohorts A and B did not differ, nor did Cohorts B and C. The overall model explained 1.0% of the variance (R^2^ = 0.010) (Figure 2, Table A2).

#### 3.1.2. Cohort Comparison of BMI and BMI *z* Score Considering Social Index (Cohorts A and C)

A two-factor analysis of variance for the dependent variable BMI revealed the effect of the interaction between cohort and social index (*p* = 0.040, n_p_^2^ = 0.004). Within Cohort A, children with a high social burden had 3.9% higher BMI values than those with low social burdens (+0.7 (0.3) kg/m^2^, *p* = 0.017, n_p_^2^ = 0.006); in Cohort C, the BMI was 8.8% higher among children with a high social burden (+1.5 (0.3) kg/m^2^, *p* < 0.001, n_p_^2^ = 0.024). Children with high social burdens only had 5.3% higher BMI values in Cohort C compared with Cohort A (+0.9 (0.2) kg/m^2^, *p* < 0.001, n_p_^2^ = 0.015). The overall model explained 8.4% of the variance (*p* < 0.001, R^2^ = 0.84) (Figure 2, Table A3 and Table A4 Model 1).

**Figure 3:**
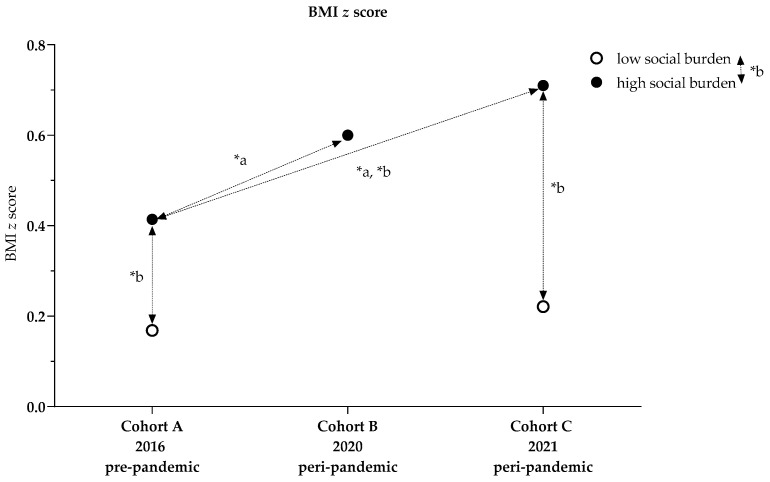
BMI *z* score comparisons between cohort and influenced by social index; * a *p*-values ANOVA, Table A2; * b *p*-values two-factor analysis adjusted for social index, Table A3 and Table A4, Model 2.

Two-factor analysis of variance for the dependent variable BMI *z* score showed that children with high social burdens had BMI *z* scores 0.4 (1.0) higher than those with a low social burden (*p* < 0.001, n_p_^2^ = 0.024). Within Cohort A, the BMI *z* scores for children with a high social burden were 0.2 (1.0) higher than for those with low social burdens (*p* = 0.013, n_p_^2^ = 0.006); in Cohort C, the average BMI *z* score was higher by 0.5 (0.1) (*p* < 0.001, n_p_^2^ = 0.020). When social burden was high, BMI *z* scores were higher by 0.3 (0.1) in Cohort A compared with Cohort C (*p* < 0.001, n_p_^2^ = 0.012). The overall model explained 4.1% of the variance (*p* < 0.001, R^2^ = 0.041) (Figure 3, Table A3 and Table A4, Model 2).

### 3.2. Motor Performance

#### 3.2.1. Lateral Jumping–Descriptive Presentation

With a high social burden, lateral jumping performance decreased from 51.3 (17.0) jumps in Cohort A to 47.1 (14.4) jumps in Cohort B and 42.3 (17.5) jumps in Cohort C (Table 1).

With a low social burden, the number of lateral jumps was 55.4 (0.9) jumps in Cohort A and 51.2 (17.0) jumps in Cohort C (Table 1).

##### Cohort Comparisons of Lateral Jumping (High Social Burden)

**Figure 4:**
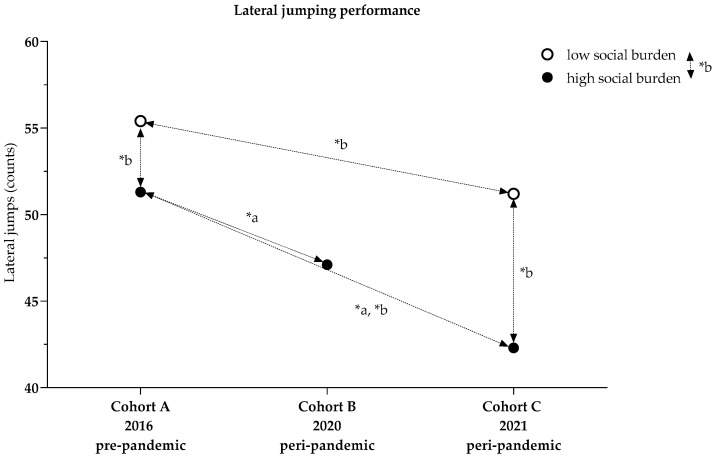
Lateral jump performance comparisons between cohort and influenced by social index; * a *p*-values ANCOVA adjusted for BMI, age, sex, Table A2; * b *p*-values two-factor analysis adjusted for social index, BMI, age, sex, Table A3 and Table A4, Model 3.

The univariate analysis of variance showed that children in Cohort B performed 7.6% less well in lateral jumping than Cohort A (−3.8 (1.3) jumps, *p* = 0.012), and children in Cohort C performed 12.3% less well than Cohort A (−6.1 (1.1) jumps, *p* < 0.001). No differences were identified between Cohorts B and C . BMI (*p* < 0.001, n_p_^2^ = 0.037) and age (*p* < 0.001, n_p_^2^ = 0.324) were influential, but sex was not. The overall model explained 35.8% of the variance (R^2^ = 0.358) (Figure 4, Table A2).

##### Cohort Comparison of Lateral Jumping Considering Social Index (Cohorts A and C)

The two-factor analysis of variance revealed that lateral jumping performance by children with high social burdens was 9.6% lower than among children with low social burdens (−4.5 (1.0) jumps, *p* < 0.001, n_p_^2^ = 0.021). In Cohort A, the performance of children with high social burdens was 7.8% lower than that of children with low social burdens (−4.3 (1.3) jumps, *p* < 0.001, n_p_^2^ = 0.010). In Cohort C, the performance of children with a high social burden was 9.8% lower (−4.8 (1.4) jumps, *p* < 0.001, n_p_^2^ = 0.011). The performance of children with a high social burden was 11.9% lower in Cohort C than in Cohort A (−6.0 (1.1) jumps, *p* < 0.001, n_p_^2^ = 0.028). Among children with a low social burden, performance was 10.0% lower in Cohort C than in Cohort A (−5.4 (1.6) jumps, *p* < 0.001 (0.012). BMI, age, and sex were influential; the overall model explained 39.0% of the variance (*p* < 0.001, R^2^ = 0.390) (Figure 4, Table A3 and Table A4, Model 3).

#### 3.2.2. Standing Long Jump–Descriptive Presentation

With a high social burden, standing long jump performance was 111.1 (22.0) cm in Cohort A, 115.2 (23.0) cm in Cohort B, and 109.2 (22.9) cm in Cohort C (Table 1).

With a low social burden, performance decreased from 123.3 (22.0) cm in Cohort A to 121.2 (25.1) cm in Cohort C (Table 1).

##### Cohort Comparisons of Standing Long Jump (High Social Burden)

**Figure 5:**
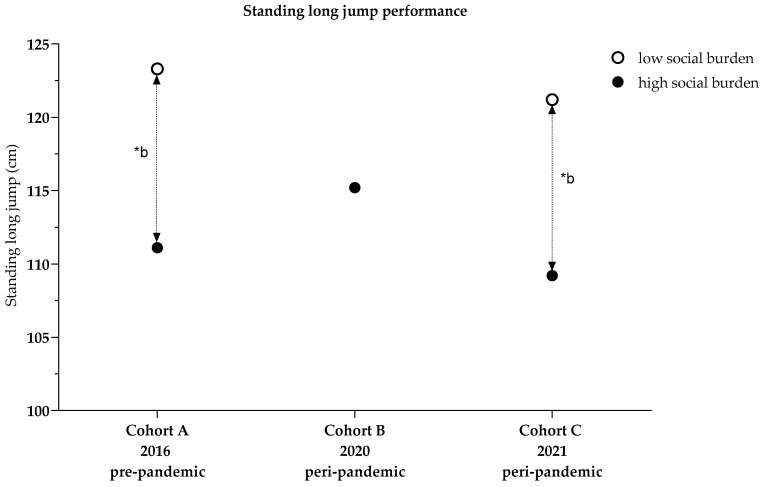
Standing long jump performance comparisons between cohorts and influenced by social index; * b *p*-values two-factor analysis adjusted for social index, BMI, age, sex, Table A3 and Table A4, Model 4.

A univariate analysis of variance revealed differences in the extent to which cohorts (*p* < 0.001) were influenced by BMI (*p* < 0.001, n_p_^2^ = 0.094), age (*p* < 0.001, n_p_^2^ = 0.206), and sex (*p* < 0.001, n_p_^2^ = 0.038). Pairwise comparisons showed a 5.4% better performance in the standing long jump in Cohort B than in Cohort A (5.8 (1.9) cm, *p* = 0.008), though no further differences were found. The overall model explained 26.9% of the variance (R^2^ = 0.269) (Figure 5, Table A2).

##### Cohort Comparison of Standing Long Jump Considering Social Index (Cohorts A and C)

A two-factor analysis of variance estimated an effect of the interaction of cohort and social index (*p* = 0.022, n_p_^2^ = 0.005). In Cohort A, children with a high social burden showed a 10.0% worse performance than children with low social burdens (−12.1 (1.9) cm, *p* < 0.001, n_p_^2^ = 0.040). In Cohort C, the performance of children with a high social burden was 4.8% lower than that of children with low social burdens (−5.7 (2.1) cm, *p* = 0.006, n_p_^2^ = 0.008). BMI, age, and sex were influential; the overall model explained 30.4% of the variance (*p* < 0.001, R^2^ = 0.304) (Figure 5, Table A3 and Table A4, Model 4).

##### 6-min Run–Descriptive Presentation

Children with a high social burden in Cohort A ran 879.3 (111.0) m on the 6-min run, while children with a high social burden in Cohort C ran 757.9 (133.2) m (Table 1).

Meanwhile, children with a low social burden in Cohort A managed a distance of 936.4 (113.6) m, while children with a low social burden in Cohort C managed 884.3 (132.2) m (Table 1).

##### Cohort Comparisons of 6-min Run (High Social Burden)

**Figure 6:**
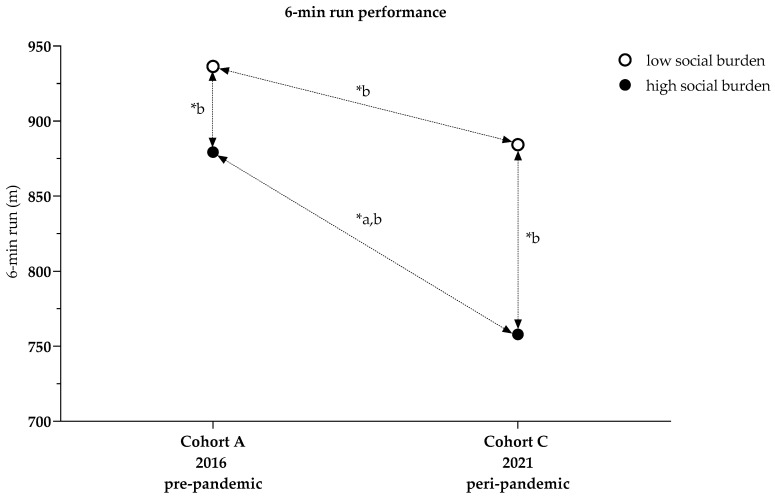
The 6-min run performance comparisons between cohorts and influenced by social index; * a *p*-values ANCOVA adjusted for BMI, age, sex, Table A2; * b *p*-values two-factor analysis adjusted for social index, BMI, age, sex, Table A3 and Table A4, Model 5.

A univariate analysis of variance revealed significant differences between Cohorts A and C (*p* < 0.001). In Cohort C, the 6-min run performance was 11.3% lower than in Cohort A (−97.4 (8.8) m, *p* < 0.001). BMI (*p* < 0.001, n_p_^2^ = 0.159), age (*p* < 0.001, n_p_^2^ = 0.117), and sex (*p* < 0.001, n_p_^2^ = 0.035) were influential. The overall model explained 36.6% of the variance (R^2^ = 0.366) (Figure 6, Table A2).

##### Cohort Comparison of 6-min Run Considering Social Index (Cohorts A and C)

Two-factor analysis of variance showed that cohort and social index interacted in such a way as to influence the 6-min running performance (*p* = 0.009, n_p_^2^ = 0.007). In Cohort A, the performance of children with high social burdens was 5.6% lower than that of children with low social burdens (−52.0 (10.2) m, *p* < 0.001, n_p_^2^ = 0.025). Likewise, in Cohort C, the performance of children with a high social burden was 10.6% lower (−91.4 (11.1) m, *p* < 0.001, n_p_^2^ = 0.062). The performance of children with a high social burden in Cohort C was 11.1% lower than that of children in Cohort A (96.8 (8.7) m, *p* < 0.001, n_p_^2^ = 0.108). Likewise, the performance of children with a low social burden in Cohort C was 6.2% lower than that of children in Cohort A (57.5 (12.2) m, *p* < 0.001, n_p_^2^ = 0.021). BMI, age, and sex were influential; the overall model explained 42.6% of the variance (*p* < 0.001, R^2^ = 0.426) (Figure 6, Table A3 and Table A4, Model 5).

## 4. Discussion

This is one of the first studies in Germany to analyze anthropometric data and the motor performance of primary school children in different cohorts, comparing results before (2016) and during (2020 and 2021) the COVID-19 pandemic and considering social factors. In both social index groups, there was a negative trend in BMI/BMI *z* scores as well as in motor performance in cohort comparisons over time. However, this trend was inconsistent with regard to the standing long jump and among children with a low social burden. In fact, the trend was only significant in the areas of lateral jumping and the 6-min run. Primary school children with high social burdens had significantly worse baseline scores than did children with low social burdens in all cohorts, meaning they were more often overweight/obese or underweight, had significantly higher BMI or BMI *z* scores, and scored lower on the motor tests. In this group, the negative trend over time was also far more pronounced and affected all parameters, although performance in the standing long jump was not significantly affected. The extent to which this result is due to the pandemic and its associated restrictions rather than being an expression of the secular trend [4,5] can only be theorized. However, our results are consistent with comparable international studies that have also found increases in body weight or BMI [25,27,28] and decreases in fitness [23,24] in children, and attributed these findings to the pandemic, thus strengthening our hypothesis that the pandemic has negatively affected children’s anthropometry and physical fitness. Schlag et al. [5] analyzed data from a cohort of more than 3000 children between 6 and 13 years in Germany who were also tested with the DKT. While the data from our group of children with a low social burden showed higher scores related to the three motor test items compared to the data of Schlag et al. [5], children with a high social burden had already scored lower in lateral jumping and standing long jump in 2016 (prior to the pandemic), but not in the 6-min run. During the pandemic, the motor test scores for lateral jumping and the 6-min run were below the 2016 baseline level in both Cohort B (2020) and Cohort C (2021).

This reduction in motor performance was particularly evident under pandemic conditions in children with a high social burden, where the values were significantly lower than those described by Schlag et al. [5] from 2018 to 2020. However, in the group with a low social burden, the values for the standing long jump and 6-min run in 2021 were higher than those described by Schlag et al. [5], as they were in 2016, and those for lateral jumping were only marginally lower.

A peculiarity was the progression of performance in the standing long jump, which initially increased in Cohort B (2020) but fell back below the baseline level (2016) in Cohort C (2021) in the high burden group. However, the score of Cohort B was still below the 2018 to 2020 Cohort score of Schlag et al. [5]. Basterfield et al. [25] also noted an increase in performance in the standing long jump over the course of the pandemic, with a concomitant decrease in endurance performance in the 20-m-shuttle-run test. Hence, for the test item standing long jump (power), we cannot confirm a pandemic effect at this point due to the heterogeneous variation.

Possibly, the altered exercise opportunities during a lockdown due to the elimination of organized sports activities in school and leisure time [7] lead primarily to a reduction in endurance performance, rather than to a reduction in other muscular fitness components, such as power (standing long jump) or agility/coordination (lateral jumping). For example, in their review, Paterson et al. [37] found a decline in outdoor activity with a concurrent increase in indoor activity—although current data suggest that indoor play is less active but more sedentary, particularly in the context of a low SES [11,17,18,19].

However, other studies, such as Ostermeier et al. [14], who surveyed Canadian parents of elementary school children, did not find an association between decreased physical activity during the pandemic and low SES in children. The authors argued that the baseline values of sports participation were often significantly lower than in children with higher SES and that access to free sports and physical activity would be a more promising alternative due to their already low participation in organized sports activities [14].

Schmidt et al. [11] found the decline in physical activity among children to be independent of families’ SES during the first lockdown. They identified families’ housing situations as a meaningful predictor of the increase or decrease in regular physical activity. Other studies conducted during the pandemic confirmed that participation in outdoor activities depends on the residential environment [9,10,38]. In general, safe environments, attractive playgrounds, and parks are key factors for influencing children’s physical activity behavior [39]. However, these conditions are found much less frequently in socially deprived areas.

Here arises a need for action, especially in socially deprived areas, to create incentives for more physical activity. This is because reduced physical activity, often in conjunction with increased sedentary behavior as well as an unhealthy diet, causes an increase in BMI [26]. The association of a high BMI with reduced motor performance, as we also found in our data, is considered a worrisome risk factor for the development of a cardiovascular risk profile [2,25].

These findings underscore the need for increased collaboration among various sectors of children and youth advocates to create comprehensive opportunities for children to engage in physical activity [7]. Promoting physical activity in schools and neighborhoods in disadvantaged areas may be one way to combat the negative health consequences of the pandemic, such as increased obesity and reduced physical performance.

### Strengths and Weaknesses

In addition to taking into consideration the social burden, a strength of this study is that we collected hard data rather than depending solely on questionnaire-based surveys of weight and fitness. However, due to the unanticipated pandemic-related restrictions, we were unable to collect data from a control group. Therefore, the results can only be attributed to the pandemic to a limited extent. In addition, the cohort comparison period was relatively long, extending from 2016 to 2020/21. However, comparisons with other data from studies conducted before the pandemic appear to corroborate our findings [5].

One limitation relates to Cohort B, which, as described in the methodology section, comprised only children from schools with high social burdens and for which data on the 6-min run were not available. Nevertheless, we included this cohort because we wanted to include as many study time points of the pandemic as possible. Because it is difficult to ascertain how individual interventions or the duration of the pandemic may have affected children, we considered it important to include the different study time points.

Another limitation relates to the fact that the data originates from two different projects. Thus, no longitudinal comparisons could be conducted, only a comparison between independent cohorts. However, both projects were carried out in the Cologne area at semi-adjoining schools; thus, there was a certain uniformity of local conditions. Likewise, data collection was based on identical methods. The test items were taken from a well-validated test battery (DKT) [5]. A major advantage of the test items selected is their high comparability with data from other studies; all three test items can be found in studies conducted in Germany and internationally for the measurement of motor performance (6-min run, e.g., [24]; standing long jump, e.g., [23,40]; lateral jumping, e.g., [41]).

Furthermore, we used the BMI *z* score for age and sex adjustment calculated by the German procedure by Kromeyer-Hauschild [32] because of the national comparability.

Although we examined changes in BMI values/BMI *z* scores, we did not evaluate dietary behavior. Containment measures have also had an important impact on diet, leading to an increase in the consumption of snacks and processed foods [42]. However, we were unable to examine whether changes in eating or physical activity behavior influenced weight development because dietary information was not available in our datasets. Additionally, information regarding other sociodemographic characteristics, such as country of origin, language spoken, schooling and vocational training, occupational status, and household income was not available in our analysis [43,44,45]. As an alternative, we used the school-specific social index of North Rhine-Westphalia. This provides an average value for each school but does not exclude that the home social conditions of individual children deviate. The advantage of such an approach is that it allows researchers to acquire knowledge about the socioeconomic status of a cohort without engaging in time-consuming surveys.

## 5. Conclusions

Our analysis demonstrated that motor performance regressed under pandemic conditions, while the BMI and BMI *z* scores of children increased, along with the prevalence of obesity and being overweight, as well as being underweight. We also demonstrated that these effects were more pronounced when children faced higher social burdens. Thus, we emphasize the urgent need for action to promote health, especially among disadvantaged children from at-risk neighborhoods. A low-threshold approach could be adopted by community-based, especially school-based, interventions due to their proximity and coverage, in order to close the gap between the socially advantaged and socially disadvantaged, which has widened even further during the course of the pandemic, and to offer all children an equally accessible platform for exercise and health.

## Figures and Tables

**Table 1:** Descriptive statistics.

	Total		Cohort A 2016 Pre-Pandemic				Cohort B 2020 Peri-Pandemic		Cohort C 2021 Peri-Pandemic			
			High Burden		Low Burden		High Burden		High Burden		Low Burden	
	*n*	*Mean (SD)*	*n*	*Mean (SD)*	*n*	*Mean (SD)*	*n*	*Mean (SD)*	*n*	*Mean (SD)*	*n*	*Mean (SD)*
*N*	1238		261		210		196		441		130	
female sex %	599	48.4%	117	44.8%	107	51.0%	100	51.0%	216	49.0%	59	45.4%
male sex %	639	51.6%	144	55.2%	103	49.0%	96	49.0%	225	51.0%	71	54.6%
age (years)	1238	8.1 (1.2)	261	8.3 (1.1)	210	8.1 (1.1)	196	8.3 (1.2)	441	8.0 (1.3)	130	8.3 (1.3)
height (cm)	1238	130.9 (9.1)	261	131.3 (8.6)	210	132.2 (8.9)	196	132.3 (9.2)	441	128.9 /9.2)	130	132.1 (8.9)
body weight (kg)	1238	30.6 (8.3)	261	30.3 (7.5)	210	29.5 (6.9)	196	32.5 (9.5)	441	30.7 (9.1)	130	29.7 (6.7)
BMI (kg/m^2^)	1238	17.6 (3.2)	261	17.4 (2.8)	210	16.7 (2.4)	196	18.3 (3.6)	441	18.2 (3.6)	130	16.9 (2.3)
BMI *z* score	1238	0.5 (1.1)	261	0.4 (1.0)	210	0.2 (0.9)	196	0.6 (1.3)	441	0.7 (1.2)	130	0.2 (0.9)
**percentile %**			261		210				441		130	
underweight	51	4.1%	9	3.4%	7	3.3%	7	3.6%	19	4.3%	9	6.9%
normal weight	883	71.3%	193	73.9%	180	85.7%	130	66.3%	280	63.5%	100	76.9%
overweight	162	13.1%	31	11.9%	11	5.2%	34	17.3%	71	16.1%	15	11.5%
obese	142	11.5%	28	10.7%	12	5.7%	25	12.8%	71	16.1%	6	4.6%
social index (level)	1238	5.0 (2.1)	261	5.5 (0.5)	210	2.5 (1.5)	196	6.5 (0.5)	441	6.5 (0.5)	130	1.0 (0.0)
**motor performance**												
lateral jumping (counts)	1232	48.1 (17.3)	261	51.3 (17.0)	209	55.4 (0.9)	196	47.1 (14.4)	437	42.3 (17.5)	129	51.2 (17.0)
standing long jump (cm)	1144	114.2 (23.5)	230	111.1 (22.0)	209	123.3 (22.0)	182	115.2 (23.0)	413	109.2 (22.9)	124	121.2 (25.1)
6-min run (m)	1038	840.1 (143.8)	261	879.3 (111.0)	208	936.4 (113.6)		N.A.	439	757.9 (133.2)	130	884.3 (132.3)

## Data Availability

As assurances were given in the study information and 53 consent forms of both studies that the data would not be shared with third parties, we are not authorized to share the datasets. Data has not been published elsewhere.

## Appendix A

**Table A1:** Overview of variables * N.A. due to hygiene measures of the COVID-19 pandemic (√ = available results).

Variable	Type	Definition	Cohort A	Cohort B	Cohort C
			2016	2020	2021
Age	Metric	Participants’ age in years at the measurement point	√	√	√
Sex	Dichotomous	Participants‘ sex, 0 = girl, 1 = boy	√	√	√
Height	Metric	Participants’ body height in cm	√	√	√
Weight	Metric	Participants’ body weight in kg	√	√	√
BMI	Metric	Participants’ body mass index (measure of weight adjusted for height) in kg/m^2^	√	√	√
BMI *z* score	Metric	Participants’ BMI standard deviation score	√	√	√
BMI percentile	Categorial	Participants’ BMI category 1 = underweight, 2 = normal weight, 3 = overweight, 4 = obesity	√	√	√
Lateral jumps	Metric	Participants‘ number of jumps during 2 × 15 s, to assess agility and coordination	√	√	√
Standing long jump	Metric	Participants’ best of two tries (in cm), to assess the rapid strength of the lower extremities	√	√	√
6-min run	Metric	Distance (in meters) run by a participant in 6 min, aerobic endurance capacity	√	N.A. *	√
Social index	Metric	Social index of each participant’s school, from 1 = very low burden to 9 = very high burden; numbers were clustered as dummy variables into 0 = low social burden (numbers 1 to 5), 1 = high social burden (numbers 6 to 9)	*√*	*√*	*√*
Pandemic Situation	Categorial	Participants were screened during COVID-19 pandemic situations in 2020 or 2021; 0 = no, 1 = yes	*√*	*√*	*√*

**Table A2:** ANCOVA post hoc with Bonferroni correction for cohorts with high social burden; * adjusted for BMI, age, and sex.

	Tests of Between-Subjects Effects				Pairwise Comparisons		
	Corrected Model	BMI	Age	Sex	Cohort B-A	Cohort C-A	Cohort C-B
	*p-Value (Adjusted R^2^)*	*p-Value (n_p_^2^)*	*p-Value (n_p_^2^)*	*p-Value (n_p_^2^)*	*ΔMW (SD),* *% Change,* *p-Value (n_p_^2^)*	*ΔMW (SD),* *% Change,* *p-Value (n_p_^2^)*	*ΔMW (SD),* *% Change,* *p-Value (n_p_^2^)*
BMI (kg/m^2^)	<0.001 (0.058)	N.A.	<0.001 (0.044)	0.362 (0.001)	Δ 0.8 (0.3) +4.9% 0.020	Δ 0.9 (0.3) +5.4% <0.001	Δ 0.1 (0.3) +0.5% 1.0
BMI *z* score	0.005 (0.010)	N.A.	N.A.	N.A.	Δ 0.2 (0.1) +48.1% 0.070	Δ 0.3 (0.1) +71.5% <0.001	Δ 0.1 (0.1) +15.8% 0.333
lateral jumps (counts) *	<0.001 (0.358)	<0.001 (0.037)	<0.001 (0.324)	0.227 (0.002)	Δ −3.8 (1.3) −7.6% 0.012	Δ −6.1 (1.1) −12.3% <0.001	Δ −2.4 (1.2) −5.1% 0.141
standing long jump (cm) *	<0.001 (0.269)	<0.001 (0.094)	<0.001 (0.206)	<0.001 (0.038)	Δ 5.8 (1.9) +5.4% 0.008	Δ 2.3 (1.6) +2.1% 0.497	Δ −3.6 (1.7) +3.1% 0.124
6-min run (m) *	<0.001 (0.366)	<0.001 (0.159)	<0.001 (0.117)	<0.001 (0.035)	N.A.	Δ −97.4 (8.8) −11.3% <0.001	N.A.

**Table A3:** *Modelling for* ANCOVA of Cohorts A vs. C, to calculate the influence of social burden (high burden vs. low burden) (see Table A4 for results).

	Dependent Variable	Factors	Covariates
Model 1	BMI	cohort, social index	age, sex
Model 2	BMI *z* score	cohort, social index	
Model 3	lateral jumps	cohort, social index	BMI, age, sex
Model 4	standing long jumps	cohort, social index	BMI, age, sex
Model 5	6-min run	cohort, social index	BMI, age, sex

**Table A4:** ANCOVA results of the models from Table A3.

Tests of Between-Subjects Effects				Pairwise Comparisons				
	Corrected Model	Cohort	Social Index	High-Low Burden	Cohort A: High-Low Burden	Cohort C: High-Low Burden	High Burden: Cohort C-A	Low Burden: Cohort C-A
	*p-Value* *(Adjusted R^2^)*	*p-Value (n_p_^2^)*	*p-Value (n_p_^2^)*	*ΔMW (SD),* *% Change,* *p-Value (n_p_^2^)*	*ΔMW (SD),* *% Change,* *p-Value (n_p_^2^)*	*ΔMW (SD),* *% Change,* *p-Value (n_p_^2^)*	*ΔMW (SD),* *% Change,* *p-Value (n_p_^2^)*	*ΔMW (SD),* *% Change,* *p-Value (n_p_^2^)*
**Model 1**	<0.001 (0.084)	0.040 (0.004) *		Δ 1.1 (0.2) +6.4% <0.001 (0.027)	Δ 0.7 (0.3) +3.9% 0.017 (0.006)	Δ 1.5 (0.3) +8.8% <0.001 (0.024)	Δ 0.9 (0.2) +5.3% <0.001 (0.015)	Δ 0.1 (0.3) +0.6% 0.769 (0)
**Model 2**	<0.001 (0.041)	0.016 (0.006)	<0.001 (0.024)	Δ 0.4 (0.1) <0.001 (0.024)	Δ 0.2 (0.1) 0.013 (0.006)	Δ 0.5 (0.1) <0.001 (0.020)	Δ 0.3 (0.1) <0.001 (0.012)	Δ 0.1 (0.1) 0.657 (0)
**Model 3**	<0.001 (0.390)	<0.001 (0.035)	<0.001 (0.021)	Δ −4.5 (1.0) −9.6% <0.001 (0.021)	Δ −4.3 (1.3) −7.8% <0.001 (0.010)	Δ −4.8 (1.4) −9.8% <0.001 (0.011)	Δ −6.0 (1.1) −11.9% <0.001 (0.028)	Δ −5.4 (1.6) −10,0% <0.001 (0.012)
**Model 4**	<0.001 (0.304)	0.022 (0.005) *		Δ −8.9 (1.4) −7.4% <0.001 (0.040)	Δ −12.1 (1.9) −10.0% <0.001 (0.040)	Δ −5.7 (2.1) −4.8% 0.006 (0.008)	Δ −2.6 (1.6) +2.4% 0.108 (0.003)	Δ −3.8 (2.3) −3.1% 0.096 (0.003)
**Model 5**	<0.001 (0.426)	0.009 (0.007) *		Δ −71.7 (7.6) −8.0% <0.001 (0.080)	Δ−52.0 (10.2) −5.6% <0.001 (0.025)	Δ −91.4 (11.1) −10.6% <0.001 (0.062)	Δ −96.8 (8.7) −11.1% <0.001 (0.108)	Δ −57.5 (12.2) −6.2% <0.001 (0.021)

* Significant interaction cohortsocial index.
